# Investigating the Effectiveness of Brexpiprazole in Subjects with Schizophrenia Spectrum Illness and Co-Occurring Substance Use Disorder: A Prospective, Multicentric, Real-World Study

**DOI:** 10.3390/ph17040535

**Published:** 2024-04-21

**Authors:** Stefania Chiappini, Clara Cavallotto, Alessio Mosca, Francesco Di Carlo, Tommaso Piro, Giulia Giovannetti, Arianna Pasino, Mariachiara Vicinelli, Chiara Lorenzini, Mariapia Di Paolo, Maria Pepe, Marco Di Nicola, Valerio Ricci, Mauro Pettorruso, Giovanni Martinotti

**Affiliations:** 1 School of Medicine, UniCamillus International Medical School University, Via di S. Alessandro 8, 00131 Rome, Italy; stefaniachiappini9@gmail.com; 2 Department of Neurosciences, Imaging and Clinical Sciences, Università degli Studi G. D‘Annunzio, 66100 Chieti, Italy; alessio.mosca909@gmail.com (A.M.); francesco.dic@hotmail.it (F.D.C.); tommasopiro19@gmail.com (T.P.); giovannettigiulia@libero.it (G.G.); pasino.arianna@gmail.com (A.P.); mchiaravicinelli@gmail.com (M.V.); lorenzini.chiara94@gmail.com (C.L.); mariapiadipaolo@yahoo.it (M.D.P.); mauro.pettorruso@hotmail.it (M.P.); giovanni.martinotti@gmail.com (G.M.); 3 University Polyclinic Foundation “A. Gemelli” IRCCS, Catholic University of the Sacred Heart, 00136 Rome, Italy; mariapepe.992@gmail.com (M.P.); marco.dinicola@policlinicogemelli.it (M.D.N.); 4 Department of Psychiatry, “San Luigi Gonzaga” Hospital, University of Turin, 10124 Turin, Italy; v.ricci@sanluigi.piemonte.it

**Keywords:** dual disorders, substance use disorder, brexpiprazole, real-world study, addiction, substance-induced psychosis

## Abstract

Background: Dual disorders (DDs) involve the coexistence of a substance use disorder (SUD) with another mental illness, often from the psychotic and affective categories. They are quite common in clinical practice and present significant challenges for both diagnosis and treatment. This study explores the effectiveness of brexpiprazole, a third-generation antipsychotic, in an Italian sample of individuals diagnosed with schizophrenia spectrum disorder and a comorbid SUD. Methods: Twenty-four patients, diagnosed according to the *Diagnostic and Statistical Manual of Mental Disorders, Fifth Edition* (*DSM-5*) and enrolled in several Italian hospitals, underwent a psychometric assessment at baseline (T0) and one month (T1) after starting brexpiprazole treatment administered at a mean dosage of 2 mg/day. Results: Brexpiprazole demonstrated significant reductions in psychopathological burden (Positive and Negative Syndrome Scale/PANSS total score: *p* < 0.001). Positive (*p* = 0.003) and negative (*p* = 0.028) symptoms, substance cravings (VAS craving: *p* = 0.039), and aggression (MOAS scale: *p* = 0.003) were notably reduced. Quality of life improved according to the 36-item Short Form Health Survey (SF-36) subscales (*p* < 0.005). Conclusions: This study provides initial evidence supporting brexpiprazole’s efficacy and safety in this complex patient population, with positive effects not only on psychopathology and quality of life, but also on cravings. Further studies involving larger cohorts of subjects and extended follow-up periods are needed.

## 1. Introduction

### 1.1. Dual Disorders

Dual disorders (DDs) are characterized by the coexistence of at least one substance use disorder (SUD) with another mental illness, where frequently, the mental illnesses involved belongs to the psychotic and affective clinical categories [1]. SUDs compass 10 specific categories of substances, as outlined in the *Diagnostic and Statistical Manual of Mental Disorders, Fifth Edition* (*DSM-5*): alcohol, caffeine, cannabis, hallucinogens, inhalants, opiates, sedatives, hypnotics, anxiolytics, and stimulants (such as amphetamines and cocaine), as well as tobacco [2]. The core characteristic of an SUD is a set of cognitive, behavioral, and physiological symptoms that demonstrate the individual’s persistent substance use despite encountering significant associated issues. These symptoms may include consuming the substance in larger amounts than intended, making multiple unsuccessful attempts to reduce or cease its use, and experiencing intense cravings [2].

The initial recognition of DDs dates back to the 1980s, with their frequent identification beginning in the 1990s. Since then, various definitions have emerged, yet a universally accepted, unambiguous definition remains elusive. Currently, the World Health Organization (WHO) defines them as “the co-occurrence in the same individual of a psychoactive substance use disorder and another psychiatric disorder,” reflecting the complexity and the overlapping nature of these conditions [3,4]. DDs present a formidable challenge for healthcare professionals, primarily due to the complex and variable interaction between psychoactive substances and psychiatric disorders. The intricacy of this interaction is amplified by the diverse effects of different substances and the wide range of mental disorders, each impacting clinical outcomes in distinct ways. This comorbidity also introduces significant behavioral complexities in patients, adding layers to the already intricate task of clinical management. Adding to this complexity is the bidirectional causality inherent in DDs. Substance misuse can lead to the development of mental illness, while mental illness can trigger substance misuse. Furthermore, there is a dynamic and mutual aggravation between the two disorders, with each potentially worsening the severity and progression of the other [5]. Three potential situations can be contemplated:

- Drug use can induce individuals to undergo one or more symptoms of a mental health disorder, either of a short-lived nature (e.g., amphetamine-induced psychosis) or by triggering an underlying long-term mental disorder (e.g., cannabis and schizophrenia);
- Mental disorders might prompt drug use as a means to alleviate the symptoms associated with the mental disorder (e.g., using amphetamines to alleviate symptoms of depression);
- Both the issue of substance use and the presence of a mental health disorder may stem from shared factors, such as brain deficits, genetic susceptibility, and early exposure to stress or trauma [6].

Furthermore, the combination of substance use and mental health disorders often leads to serious social, psychological, and physical complications. Consequently, psychiatric comorbidity significantly contributes to the global burden of diseases, particularly among vulnerable population groups. Secondly, individuals with comorbid disorders are less frequently diagnosed, have reduced access to care, and demonstrate lower treatment compliance [6]. Definitively, individuals experiencing psychosis typically demonstrate a higher prevalence of problematic alcohol consumption and illicit drug use compared to the general population [7]. While ongoing research seeks to establish a definitive causal relationship between illicit drug use and the onset of psychosis [8], there is a consensus regarding the harmful effects of substance misuse on the course of psychosis. This influence leads to a more prolonged and severe psychopathology, impacting various aspects of an individual’s life, including non-compliance with prescribed medications, inadequate engagement with treatment programs, a heightened risk of suicide, an increased frequency of hospitalizations, an elevated likelihood of involvement in violent incidents, extended periods within the criminal justice system, and an overall compromised prognosis [9,10]. Although the complexity of this condition is clear, the current edition of the *Diagnostic and Statistical Manual of Mental Disorders* (*DSM-5*) does not provide specific diagnostic criteria for DDs as co-occurring conditions [2]. The lack of standardized definitions and diagnostic criteria for DDs in authoritative texts like the *DSM-5* thus hinders the accurate determination of its prevalence.

### 1.2. Dual Disorders: What Treatments Are Available?

The prevalence of DDs varies depending on the population under study and the criteria used for their classification. In the United States, according to estimates from the Substance Abuse and Mental Health Services Administration (SAMHSA), nearly half of young adults aged 18 to 25 in 2021 (45.8% or 15.3 million individuals) experienced either an SUD or any mental illness within the past year [11]. This percentage was higher compared to corresponding figures among adults aged 26 to 49 (39.5% or 40.4 million individuals) and adults aged 50 or older (22.6% or 26.7 million individuals) [12]. Unfortunately, there is a lack of available data on dual diagnoses in Italy. However, a recent study examined the prevalence of concurrent disorders among individuals with an SUD in a specific rural area in Northern Italy. The study found that out of 750 patients surveyed, 24% had a DD, and among them, only 46.1% had received treatment through an integrated clinical program. Specifically, the majority (42.8%) of these individuals were dependent on central nervous system depressants such as heroin and cannabis. On the other hand, the most common mental illnesses identified were mood disorders (40%), personality disorders (33.3%), and schizophrenia or other psychotic disorders (13.3%) [13].

Various studies document the rising prevalence of DDs [14], wherein social changes and external factors, such as the coronavirus disease (COVID) pandemic [15] and environmental changes [16], can additionally serve as triggering elements. Among the psychopathological effects associated with substance use, psychosis stands out as particularly significant, especially when related to the use of cannabis [17] and of new/novel psychoactive substances (NPSs) [18]. In recent years, there has been a proliferation of various NPS, including synthetic cannabinoids, cathinone derivatives, psychedelic phenethylamines, novel stimulants, synthetic opioids, tryptamine derivatives, phencyclidine-like dissociatives, piperazines, and gamma-amino-butyric acid (GABA)-A/B receptor agonists [19]. These substances are becoming more common and available in the realm of substance abuse [20]. Although considered relatively safe by users, their use has been associated with severe psychopathological consequences, especially in vulnerable populations. Indeed, many NPSs can trigger psychotic symptoms, leading to a condition known as *substance-induced psychosis*. This condition, recognized and diagnosed according to the *DSM-5*, requires that the psychotic symptoms be transient and directly linked to the effects of the substance used. This condition is defined by the temporal nature of these symptoms, indicating that they are not expected to persist over a prolonged period [2,18]. The progression of symptoms in cases of substance-induced psychosis can be gradual, making a clear distinction between this condition and primary psychotic disorders challenging and often not practically useful.

In this regard, according to the Canadian Schizophrenia Guidelines, the optimal outcomes for cases involving comorbidity with SUDs are achieved through the concurrent use of antipsychotic medications and addiction-focused psychosocial interventions. However, there is currently insufficient evidence to support the preference of one antipsychotic medication over another or to favor one psychosocial intervention over another for individuals diagnosed with schizophrenia and other psychotic disorders, especially when accompanied by co-occurring SUDs [21]. In such scenarios, where definitive guidelines are absent, clinicians are required to base their medication choices on careful clinical observations and the specific characteristics of each individual case. This should make us understand the need to identify specific effective treatments in the context of a DD. Inizio moduloFine modulo.

In this context, real-world studies, including observational research, play a vital role in comprehending the effectiveness, safety, and tolerability of medications. Indeed, they help identify factors that influence treatment responses within diverse patient populations beyond controlled clinical trial environments. This encompasses specific patient groups such as the elderly, pediatric patients, or those with comorbid medical conditions or SUDs. Real-world investigations focusing on the use of antipsychotics in patients with comorbid substance use are still limited but steadily expanding. Initial studies have indicated that atypical antipsychotics like clozapine, risperidone, and olanzapine have demonstrated effectiveness in managing psychotic symptoms and reducing substance use in individuals with DDs [22,23,24,25,26]. Specifically, according to recent studies, clozapine treatment is linked to markedly higher chances of maintaining abstinence from substance use and a reduced probability of psychiatric hospitalization in comparison to continued treatment with other antipsychotic medications [27,28]. This reduction in the intensity of craving could be mediated by the intrinsic characteristics of clozapine. On one hand, it exhibits selective binding and occupies relatively low levels of dopamine type 2 receptors in the striatum. On the other hand, it enhances inhibitory neurotransmission mediated by GABA-B and decreases glutamate levels [29,30]. Nevertheless, several side effects are often reported, including increased weight, scialorrea, white blood cell count changes, etc. Moreover, other investigations have produced varied results, suggesting that the efficacy of treatment may be affected by individual patient attributes and the specific subtype of the SUD [31,32,33,34]. This emphasizes the importance of nuanced clinical judgment and individualized patient care in the management of these complex conditions.

### 1.3. The Potential Role of Brexpiprazole in Dual Disorders

As previously mentioned, the utilization of atypical antipsychotics can be particularly beneficial in DDs, given their dual efficacy in addressing both psychotic and affective symptoms. This characteristic makes them a versatile option for treating conditions where both sets of symptoms are present or intertwined [4,35]. Brexpiprazole, classified as a third-generation antipsychotic, is characterized by its partial agonist activity at D2 receptors, a defining feature of its pharmacological profile within this class of medications. Due to its partial agonist action at D2 receptors, brexpiprazole can modulate dopaminergic activity in the dorsolateral prefrontal cortex (DLPFC), thus enhancing dopaminergic neurotransmission at low doses, while, conversely, at higher doses, acting to block dopaminergic activity [36]. Within the therapeutic dosage range, brexpiprazole binds to 59–75% of dopaminergic receptors, a proportion similar to older-generation antipsychotics. This substantiates its efficacy in addressing positive psychotic symptoms [37]. Moreover, brexpiprazole exhibits an intrinsic activity level in response to D2 receptors like cariprazine or aripiprazole, indicating that it is predominantly antagonistic. However, compared to both older-generation antipsychotics and aripiprazole, it exhibits a lower incidence of adverse effects (AE) such as akathisia, nausea, or vomiting [38].

### 1.4. Aim of the Study

The main objective of our research is to assess the efficacy of brexpiprazole in patients with schizophrenia comorbid with an SUD. Secondarily, this study will address the safety and tolerability of brexpiprazole in schizophrenic patients with a concurrent SUD, focusing on the risk of side effects.

## 2. Results

### 2.1. Baseline Characteristics

A total of 24 subjects were enrolled (M: 19/F: 5; mean age: 29.4 ± 7.5); a comprehensive presentation of their sociodemographic and clinical information is detailed in Table 1.

The most common psychiatric diagnosis within our sample was substance-induced psychosis (*n* = 18; 75%), followed by schizoaffective disorder (*n* = 6; 25%), while the prevailing coexisting diagnosis was personality disorder (*n* = 4; 16.7%).

Concerning the diagnosis of SUDs, the predominant substances involved were recorded in the following order: cocaine (*n* = 11; 45.8%), alcohol (*n* = 9; 37.5%), cannabis (*n* = 11; 45.8%), methamphetamine (*n* = 1; 4.2%), ketamine (*n* = 1; 4.2%), and NPS (*n* = 1; 4.2%). Significantly, a substantial proportion of the participants were characterized as polysubstance users (*n* = 10; 41.7%), emphasizing the gravity of the situations of the patient population investigated in this study.

Regarding the pharmacological treatment, most subjects (*n =* 17; 70.8%) were antipsychotic drug-free, so they were immediately initiated on brexpiprazole. Specifically, four of these patients (16.7%) were exclusively treated with brexpiprazole throughout the study period. Conversely, seven patients (29.2%) were already on antipsychotic therapy; therefore, they underwent a cross-tapering process with brexpiprazole. Specifically, two participants (8.3%) were receiving olanzapine ranging from 15 to 20 mg/day, two (8.3%) were using promazine ranging from 40 to 100 mg/day, and one (4.2%) was undergoing treatment with quetiapine at a dosage of 100 mg/day.

In terms of other medications prescribed apart from brexpiprazole, seven patients (29.2%) were taking antidepressants; in particular, three patients (12.5%) were taking trazodone ranging from 50 to 220 mg/day, two (8.3%) were using sertraline at 50 mg/day, one (4.2%) was on paroxetine at 20 mg/day, and one (4.2%) was undergoing therapy with vortioxetine at 10 mg/day.

Furthermore, in conjunction with brexpiprazole therapy, 15 patients (62.5%) were taking mood stabilizers as follows: six (25%) were receiving valproate at a dosage ranging from 600 to 1500 mg/day, one (4.2%) was using lamotrigine at 300 mg/day, four (16.7%) were undergoing treatment with gabapentin at a dosage ranging from 900 to 1600 mg/day, two (8.3%) were using pregabalin at a dosage ranging from 150 to 450 mg/day, one (4.2%) was receiving lithium sulfate at 83 mg/day, and one (4.2%) was receiving lithium carbonate at 600 mg/day.

Regarding the treatment with benzodiazepines and Z-drugs, the main molecules patients were taking in addition to brexpiprazole were as follows: delorazepam ranging from 3 to 10 mg/day (*n* = 3; 12.5%), diazepam ranging from 7 to 22 mg/day (*n* = 3; 12.5%), lorazepam at 7.5 mg/day (*n* = 1; 4.2%), clonazepam at 2.5 mg/day (*n* = 1; 4.2%), and zolpidem at 10 mg/day (*n* = 1; 4.2%).

Finally, two subjects (8.3%) were taking other drugs than those previously mentioned, in addition to brexpiprazole; specifically, one patient (4.2%) was taking methadone at 55 mg/day and one patient (4.2%) was using baclofen 35 mg/day.

### 2.2. Changes in Psychopathological Domains from Baseline to One-Month Follow-Up

**Figure 1:**
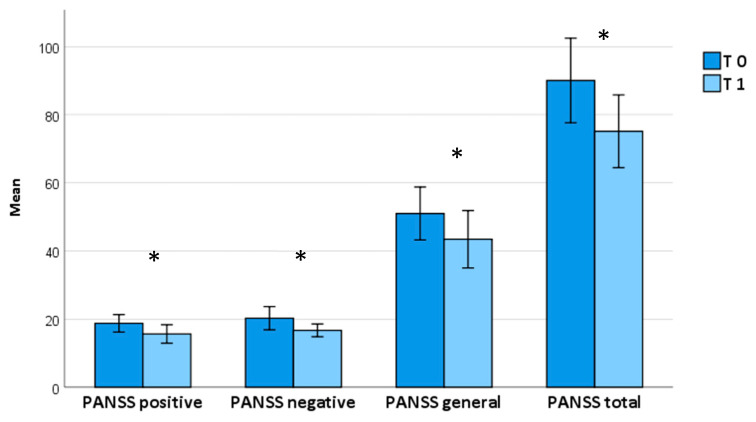
PANSS scores from baseline to one-month follow-up. *Abbreviations:* PANSS: Positive and Negative Syndrome Scale. Bars represent means ± 2 SEs. * means that it is statistically significant.

**Figure 2:**
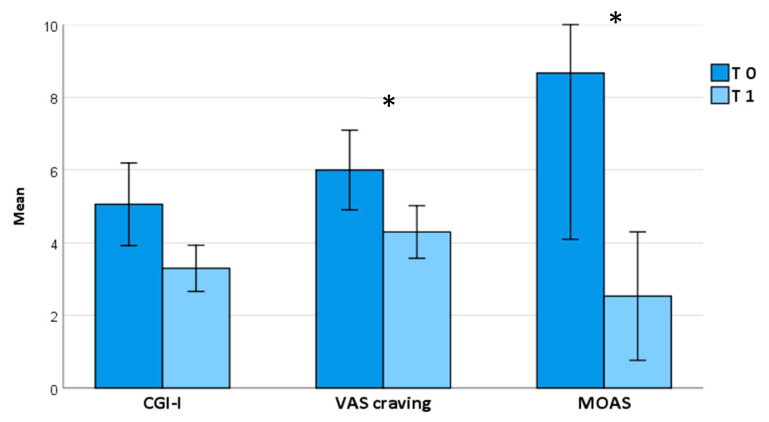
GCI-I, VAS craving, and MOAS scores from baseline to one-month follow-up. *Abbreviations:* CGI-I: Clinical Global Impression Improvement; MOAS: Modified Overt Aggression Scale; VAS: visual analogue scale. Bars represent means ± 2 SEs. * means that it is statistically significant.

Brexpiprazole treatment at an average dosage of 2 mg once daily (2.3 ± 0.9) proved effective in reducing the overall psychopathological burden after one month, as evidenced by a significant decrease in the total PANSS score (T0 = 90.0 ± 30.4, T1 = 73.4 ± 21.9; *p* < 0.001) as well as its subscales (*positive symptoms*: T0 = 18.8 ± 6.3, T1 = 15.6 ± 5.4, *p* = 0.003; *negative symptoms:* T0 = 20.3 ± 8.4, T1 = 16.1 ± 4.3, *p* = 0.028; *general psychopathology*: T0 = 51.0 ± 19.0, T1 = 42.7 ± 16.6, *p* = 0.003) (Table 2; Figure 1); similarly, the total MOAS score (T0 = 8.3 ± 8.7, T1 = 2.5 ± 3.6, *p* = 0.003) and VAS craving score (T0 = 5.9 ± 2.3, T1 = 4.3 ± 1.5, *p =* 0.039) improved (Table 2; Figure 2).

### 2.3. Changes in Global Health Condition from Baseline to One-Month Follow-Up

**Figure 3:**
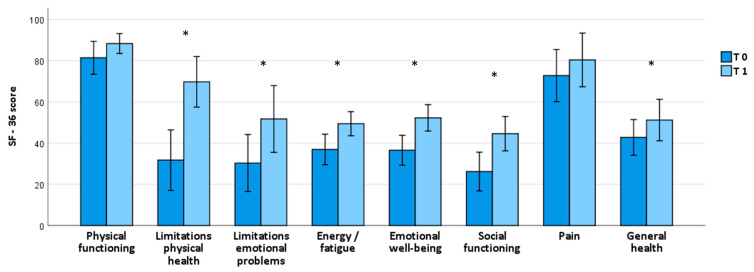
SF-36 scores from baseline to one-month follow-up. *Abbreviations*: SF-36: 36-item Short Form Health Survey. Bars represent means ± 2 SEs. * means that it is statistically significant.

A notable self-reported enhancement in global health condition was noted one month after initiating brexpiprazole treatment, as highlighted by the significant increase in some of the SF-36 subscales, e.g., *Limitations due to physical health:* T0 = 31.7 ± 35.9, T1 = 69.7 ± 24.5, *p* = 0.032; *Limitations due to emotional problems:* T0 = 30.3 ± 33.9, T1 = 51.7 ± 32.4, *p* = 0.030; *Energy/fatigue:* T0 = 36.9 ± 18.2, T1 = 49.4 ± 11.7, *p* = 0.016; *Emotional well-being*: T0 = 36.5 ± 17.7, T1 = 52.2 ± 12.8, *p =* 0.005; *Social functioning:* T0 = 26.1 ± 23.0, T1 = 44.5 ± 16.7, *p* = 0.039; and *General health*: T0 = 42.7 ± 21.2, T1 = 51.1 ± 20.1, *p =* 0.033 (Table 2; Figure 3).

### 2.4. Safety and Tolerability of Brexpiprazole

Brexpiprazole demonstrated overall high safety and good tolerability, with no side effects reported. Moreover, there were no dropouts due to drug-related side effects.

However, we observed a substantial non-adherence rate to the treatments, with 29.2% (7 out of 24) of patients discontinuing treatment prior to T1. Among these, 4.2% (one patient) relapsed into substance use, 4.2% (one patient) experienced low effectiveness in improving their psychiatric disorder, and 20.8% (five patients) dropped out due to non-adherence to drug therapy.

## 3. Discussion

This Italian study, conducted on a sample of 24 subjects with schizophrenia spectrum disorder comorbid with SUD, presents initial evidence for the use of the antipsychotic brexpiprazole in the treatment of schizophrenia comorbid with SUD, showing its efficacy, its safety, and the limitations of its use in this population.

Brexpiprazole is an atypical antipsychotic medication that is used for the treatment of schizophrenia and as an adjunctive treatment for major depressive disorder. It has a unique pharmacological profile, and its use in patients with psychotic symptoms and comorbid addiction presents both potential benefits and challenges.

In this regard, brexpiprazole demonstrates partial agonist activity at the 5HT_1A_ receptor and functions as an antagonist at the α_1_ receptor, thus resulting in a significantly reduced incidence of extrapyramidal symptoms (EPS) and akathisia [37]. In addition, the partial agonist activity of brexpiprazole at the 5HT_1A_ and 5HT_7_ receptors also imparts beneficial effects on cognitive function, mood, and anxiety [39,40].

As a result, brexpiprazole’s pharmacodynamic and pharmacokinetic profiles, on one hand, hold the promise of enhancing the effectiveness of schizophrenia treatment in dimensions where previous antipsychotics were not sufficiently effective, including depressive or cognitive symptoms [41]. On the other hand, it could also be beneficial in the treatment of mood disorders, often associated with substance abuse issues [41].

Specifically, in our study, low-dosage brexpiprazole (up to 2 mg/day) demonstrated efficacy in the treatment of psychotic symptoms and provided benefits for individuals with potential comorbid mood-related symptoms. Crucially, there was a significant reduction in substance cravings, as indicated by the VAS craving measurement, alongside a decrease in aggressiveness measured by the MOAS scale. This suggests an overall mood-stabilizing effect, enhancing quality of life. This improvement was reflected in various aspects of the SF-36 subscales, with increased scores in energy, emotional well-being, social functioning, and general health, and decreased limitations stemming from emotional issues and due to physical health. The mood-enhancing effects of brexpiprazole likely played a role in normalizing ideational content while easing initial agitation. Enhanced ideational and affective functions probably positively impacted patients’ motivation, leading to a reduction in psychopathological processes associated with substance abuse and craving. Consequently, this facilitated continued therapeutic progress and sustained abstinence from substances, with greater insight. Furthermore, the improvement in quality of life could also possibly be due to a lower incidence of side effects and a typical once-daily administration. Indeed, no dropouts due to drug-related side effects were recorded. These data are consistent with recent systematic reviews reporting brexpiprazole as a promising new drug in the pharmacotherapy of schizophrenia for both acute exacerbation and maintenance treatment, and, compared to currently used antipsychotics, for better tolerability and safety profiles [4,35].

Our findings align with a recent Italian observational study including 85 adult patients with a diagnosis of schizophrenia with either a comorbid SUD (55.8%) or no SUD (44.2%) treated with brexpiprazole at 4 mg/day for 6 months, which showed improvements over the course of the study for CGI-S, the Brief Psychiatric Rating Scale (BPRS), and PANSS in both the SUD and non-SUD groups and the entire sample, and in the SUD group, there was a statistically significant reduction in substance craving [42]. Supporting this, an animal study by Nickols et al. (2023) offered preclinical support for the effectiveness of brexpiprazole as a dopamine partial agonist in modulating behaviors dependent on dopamine during both opioid use and withdrawal, e.g., pathological drug-seeking behavior and other symptoms associated with opioid withdrawal following drug discontinuation [43].

However, we observed a high treatment dropout rate (29.2%), with 7 in 24 subjects discontinuing brexpiprazole treatment prior to one month, likely due to the inherent nature of the study. Indeed, these data can be elucidated by the intrinsic characteristics of the sample and the peculiar features of SUD, which may have influenced adherence to drug therapy (5 out of 7 patients discontinued treatment due to non-adherence to drug therapy), as evidenced by various studies on SUD [44,45,46]. On one hand, most patients were managed in an outpatient setting, which presented challenges for continuous monitoring and tracking in cases of missed appointments. On the other hand, in this study, a significant portion of the patients involved had a moderate-to-severe dual diagnosis, characterized by polysubstance abuse, heightened aggression, engagement in violent incidents, prolonged durations of involvement in the criminal justice system, and an overall unfavorable prognosis, consistent with previous research [9,10].

In this context, it is important to consider the need in DD populations for an integrated treatment approach that addresses both psychotic symptoms and addiction, with collaboration between mental health and addiction professionals being crucial. Treatment plans should be tailored to the individual’s specific needs, considering the type and severity of psychotic symptoms, the substances involved, and other relevant factors, e.g., environmental, familiar, social, personal, medical, etc. In consideration of the risk of relapse, substance use can complicate the treatment course; thus, the close monitoring of both mental health symptoms and substance use are integral components of care. Moreover, providing education to patients about the medication, its potential side effects, the importance of adherence, and the prevention of relapse in substance use is essential for treatment success. In fact, individuals with comorbid conditions may encounter difficulties in adhering to medication; in this context, brexpiprazole is typically given once daily, ensuring the essential consistency required for its effectiveness. In this regard, educating patients about the potential risks and benefits of brexpiprazole, along with the significance of adhering to the medication regimen, can improve treatment outcomes.

Ultimately, there is a need to extend and broaden the scope of rehabilitation endeavors and psychosocial interventions, transitioning them beyond residential facilities to encompass the community. This entails the establishment of an array of mental health services within the community that actively engage both patients and their family members.

### Limitations and Strengths of the Study

Despite showing important strengths, such as the multicentricity of the research conducted in several hospitals in various Italian regions, and the non-randomized nature of the research representing a real-word situation, our study encountered various limitations. Firstly, the sample size was relatively small, necessitating an expansion to enhance the reliability of our findings. A power analysis was not conducted prior to recruitment to determine the optimal sample size. Additionally, the absence of comparison groups, such as individuals treated with antipsychotics other than brexpiprazole or a placebo, hindered a comprehensive analysis. Moreover, the duration of the study was relatively limited, requiring further follow-ups; furthermore, currently, long-term data on the safety and efficacy of brexpiprazole are still evolving, and its use in certain populations may require careful consideration. An additional limitation is the lack of a standardized approach for evaluating treatment adherence. Finally, the utilization of open-label studies introduces the potential for biased results and restricts the applicability of our conclusions.

## 4. Materials and Methods

### 4.1. Participants and Recruitment Centers

In this prospective, multicentric, real-world study, a total of twenty-four patients (19 males and 5 females; mean age 29.4 ± 7.5) diagnosed with schizophrenia spectrum disorder (*DSM-5*) [2] and a comorbid SUD (*DSM-5*) [2] were consecutively recruited from several Italian mental health facilities. The coordination center was the Psychiatric Diagnostic and Treatment Service of the University Hospital S.S. Annunziata in Chieti. Other centers involved were the Inpatient Psychiatric Center of Villa Maria Pia in Rome, the Day Hospital of Psychiatry and Drug Dependence of the University General Hospital ‘A. Gemelli’ in Rome, and the Psychiatry Outpatient Clinics at the University Hospital ‘San Luigi Gonzaga’ in Turin. The Psychiatric Diagnostic and Treatment Service of the University Hospital S.S. Annunziata in Chieti is a specialized unit dedicated to the assessment and treatment of patients with psychiatric disorders presenting an acute episode of illness. Patients admitted receive a comprehensive range of interventions, including an accurate diagnosis and the implementation of personalized treatment plans. The staff may include psychiatrists, psychologists, psychiatric nurses, and other mental health professionals. Interventions may involve pharmacological therapies, in-depth psychiatric assessments, and especially continuous patient monitoring. The Inpatient Psychiatric Center of Villa Maria Pia in Rome is a specialized psychiatric treatment center designed for post-acute patients, offering a 30-day hospitalization period, extendable to a maximum of 60 days. Post-acute patients are those with elevated care requirements necessitating targeted interventions to stabilize their clinical condition following an acute episode of illness. This includes individuals discharged from the hospital psychiatric diagnostic and treatment service, or, in less severe cases than those admitted to the hospital, individuals still in need of inpatient care. The facility focuses on medication monitoring and the establishment of a medium- to long-term therapeutic program. The Day Hospital of Psychiatry and Drug Dependence of the University General Hospital ‘A. Gemelli’ in Rome and the Psychiatry Outpatient Clinics at the University Hospital ‘San Luigi Gonzaga’ in Turin engage in a range of activities aimed at the assessment, diagnosis, treatment, and care of individuals with mental health disorders and substance use disorder who do not require continuous hospitalization monitoring. These centers typically comprise a multidisciplinary team of professionals, including psychiatrists, psychologists, and nurses. Psychiatrists conduct thorough clinical assessments to diagnose various mental health conditions. This involves interviewing patients, reviewing medical histories, and employing standardized assessment tools. Additionally, these professionals prescribe and manage medications to address psychiatric symptoms, evaluating their effectiveness and monitoring any side effects.

### 4.2. Treatment Information

This study focused on a total of 24 subjects with an established diagnosis of schizophrenia spectrum and comorbid SUD, according to the *DSM-5* [2]. Subjects enrolled were carefully evaluated by qualified psychiatrists, who investigated each patient’s previously documented history of the disease and their treatment history.

Patients included in the study who had not been previously treated with antipsychotic drugs or had been free of antipsychotic medication for a minimum of 2 weeks, were initiated on brexpiprazole, following the recommended titration of 1 mg once daily to an adjustment to 2–4 mg once daily, based on the clinical response. If they were on other antipsychotic medication, patients underwent a cross-tapering process with brexpiprazole. Once the appropriate dose for each patient was reached (based on clinical progress and the clinician’s decision), the regimen was maintained for a duration of one month. Moreover, a qualified psychiatrist carefully evaluated adverse events related to brexpiprazole administration and reported them in the patients’ medical records.

Brexpiprazole’s receptor profile makes it a highly manageable medication, characterized by a low incidence of side effects: this molecule exhibits significant antagonistic action at the 5HT_2A_ receptors, aligning it closely with the pharmacodynamic properties typically associated with second-generation antipsychotics [36]. This makes it more suitable for extended use, potentially reducing the risk of hyperprolactinemia, weight gain, insomnia, nausea/vomiting, restlessness, and extrapyramidal symptoms [47].

### 4.3. Inclusion and Exclusion Criteria

The eligibility criteria for patients were as follows: an age between 18 and 65 years, with a diagnosis of schizophrenia spectrum disorder and a concurrent SUD. Patients with ECG alterations (e.g., QTc > 450 ms), an SUD in remission (more than 3 months without symptoms), acute intoxication from alcohol and substances, or severe suicidal ideation were excluded from the study. Patients were also excluded if they were pregnant or lactating and if they had a severe physical illness or evidence of mental illness severely interfering with their cognitive capacity.

### 4.4. Study Design and Psychometric Assessments

Anamnestic data were collected at baseline (T0), while psychometric assessments were collected at T0 and one month (T1) after treatment beginning. Psychiatric symptoms were evaluated using Positive and Negative Syndrome Scale (PANSS) [48] and Clinical Global Impression Improvement (CGI-I) [49] scores. The level of craving for alcohol and/or substances was evaluated by a 10 cm visual analogue scale (VAS) [50] that assesses cravings from 0 (indicating no craving) to 10 (representing the most intense craving as perceived by the patient). Furthermore, quality of life (36-item Short Form Health Survey/SF-36) [51] as well as alcohol- and substance-related aggressiveness (Modified Overt Aggression Scale/MOAS) [52] were assessed.

### 4.5. Statistical Analysis

Data analyses were performed using IBM SPSS Windows version 25. The Shapiro–Wilk test was used to determine whether the data were normally distributed. The Wilcoxon signed-rank test was used to study matched samples. In order to correct for multiple comparisons, the Benjamini–Hochberg test was used. The false discovery rate (FDR) for the Benjamini–Hochberg test was set at 0.05. The quantitative parameters are presented as means ± standard deviation (SD) and the qualitative parameters as numbers and percentages per class. The significance level was set at *p* < 0.05.

### 4.6. Ethics

All the subjects enrolled, after receiving detailed information regarding the characteristics of the drug, the prescribed dosage schedule, and the possible side effects, provided written consent with full awareness and understanding. Moreover, patients were made aware of the option to revoke their consent at any given moment.

This study was approved by the local ethics committee (protocol n. 7/09-04-2015), local institutional review boards, and the national regulatory authorities in accordance with local requirements. It was conducted in accordance with the Good Research Practice guidelines and the Declaration of Helsinki (1964) and its subsequent revisions [53].

## 5. Conclusions

This research provides a preliminary analysis of the efficacy of brexpiprazole in treating schizophrenia concurrent with SUDs. The results indicate a reduction in psychopathological burden, an improvement in quality of life, and a decrease in cravings and substance-related aggression after one month of brexpiprazole treatment. However, the high non-adherence rates highlight the challenges in managing this population, underscoring the need for personalized treatment strategies and integrated mental health services. Although this study offers initial evidence supporting the efficacy of brexpiprazole in this complex patient sample, further studies involving larger cohorts of subjects and extended follow-up periods are needed.

## Figures and Tables

**Table 1:** Demographics and clinical data of the sample (*n* = 24). Abbreviations: M: male; NPS: new/novel psychoactive substance.

**Sex, M**	19 (79.2)
**Age, years**	29.4 ± 7.5 (19–46)
**Substance abused**	
*Alcohol*	9 (37.5)
*Cocaine*	11 (45.8)
*Cannabis*	11 (45.8)
*Methamphetamine*	1 (4.2)
*Ketamine*	1 (4.2)
*NPS*	1 (4.2)
**Polysubstance users**	10 (41.7)
**Diagnosis**	
*Substance-induced psychosis*	18 (75)
*Schizoaffective disorder*	6 (25)
**Coexisting diagnosis**	
Personality disorders: - *Personality disorder NAS* - *Schizoid personality disorder* - *Schizotypal personality disorder*	4 (16.7) 2 (8.3) 1 (4.2) 1 (4.2)
**Brexpiprazole dosage (mg)**	2.3 ± 0.9 (1–4)
**Psychotropics other than brexpiprazole**	
*Antipsychotics*	Olanzapine 15–20 mg/day, 2 (8.3) Promazine 40–100 mg/day, 2 (8.3) Quetiapine 100 mg/day, 1 (4.2)
*Antidepressants*	Trazodone 50–220 mg, 3 (12.5) Sertraline 50 mg, 2 (8.3) Paroxetine 20 mg, 1 (4.2) Vortioxetine 10 mg, 1 (4.2)
*Mood stabilizers*	Valproate 600–1500 mg/day, 6 (25) Lamotrigine 300 mg/day, 1 (4.2) Gabapentin 900–1600 mg/day, 4 (16.7) Pregabalin 150–450 mg/day, 2 (8.3) Lithium sulfate 83 mg/day, 1 (4.2) Lithium carbonate 600 mg/day, 1 (4.2)
*Benzodiazepines and Z-drugs*	Lorazepam 7.5 mg/day, 1 (4.2) Clonazepam 2.5 mg/day, 1 (4.2) Delorazepam 3–10 mg/day, 3 (12.5) Diazepam 7–22 mg/day, 3 (12.5) Zolpidem 10 mg/day, 1 (4.2)
*Others*	Methadone 55 mg/day, 1 (4.2) Baclofen 35 mg/day, 1 (4.2)

Data are presented as *n* (%), means ± SDs, and ranges, as appropriate.

**Table 2:** Changes in psychometric scales from baseline to one-month follow-up. *Abbreviations:* CGI-I: Clinical Global Impression Improvement; MOAS: Modified Overt Aggression Scale; PANSS: Positive and Negative Syndrome Scale; SF-36: 36-item Short Form Health Survey; VAS: visual analogue scale.

	Baseline (*n* = 24)	Follow-Up (*n* = 17)	Z	Adjusted *p*
PANSS				
*Positive*	18.8 ± 6.3	15.6 ± 5.4	−3.292	**0.003**
*Negative*	20.3 ± 8.4	16.1 ± 4.3	−2.467	**0.028**
*General*	51.0 ± 19.0	42.7 ± 16.6	−3.433	**0.003**
*Total*	90.0 ± 30.4	73.4 ± 21.9	−3.576	**<0.001**
**CGI-I**	5.1 ± 2.4	3.3 ± 1.3	−1.610	0.122
**MOAS**	8.3 ± 8.7 7 (0–33)	2.5 ± 3.6 0 (0–12)	−3.300	**0.003**
**VAS craving**	5.9 ± 2.3	4.3 ± 1.5	−2.184	**0.039**
SF-36				
*Physical functioning*	81.3 ± 19.5	88.3 ± 9.7	−1.373	0.176
*Limitations due to physical health*	31.7 ± 35.9	69.7 ± 24.5	−2.329	**0.032**
*Limitations due to emotional problems*	30.3 ± 33.9	51.7 ± 32.4	−2.390	**0.030**
*Energy/fatigue*	36.9 ± 18.2	49.4 ± 11.7	−2.712	**0.016**
*Emotional well-being*	36.5 ± 17.7	52.2 ± 12.8	−3.044	**0.005**
*Social functioning*	26.1 ± 23.0	44.5 ± 16.7	−2.143	**0.039**
*Pain*	72.7 ± 31.0	80.3 ± 26.0	−1.355	0.176
*General health*	42.7 ± 21.2	51.1 ± 20.1	−2.281	**0.033**

Data are presented as means ± SDs and medians (ranges), as appropriate. Benjamini–Hochberg procedure was used to adjust *p*.

## Data Availability

Data is contained within the article.
